# Leukemic Stem Cells: From Leukemic Niche Biology to Treatment Opportunities

**DOI:** 10.3389/fimmu.2021.775128

**Published:** 2021-10-15

**Authors:** Tony Marchand, Sandra Pinho

**Affiliations:** ^1^ Service d’Hématologie Clinique, Centre Hospitalier Universitaire de Rennes, Rennes, France; ^2^ Faculté de médecine, Université Rennes 1, Rennes, France; ^3^ Institut National de la Santé et de la Recherche Médicale (INSERM) U1236, Rennes, France; ^4^ Department of Pharmacology & Regenerative Medicine, University of Illinois at Chicago, Chicago, IL, United States

**Keywords:** leukemic stem cell (LSC), acute myeloid leukemia, stem cell niche, genetic heterogeneity, therapeutic targets

## Abstract

Acute myeloid leukemia (AML) is one of the most common types of leukemia in adults. While complete remission can be obtained with intensive chemotherapy in young and fit patients, relapse is frequent and prognosis remains poor. Leukemic cells are thought to arise from a pool of leukemic stem cells (LSCs) which sit at the top of the hierarchy. Since their discovery, more than 30 years ago, LSCs have been a topic of intense research and their identification paved the way for cancer stem cell research. LSCs are defined by their ability to self-renew, to engraft into recipient mice and to give rise to leukemia. Compared to healthy hematopoietic stem cells (HSCs), LSCs display specific mutations, epigenetic modifications, and a specific metabolic profile. LSCs are usually considered resistant to chemotherapy and are therefore the drivers of relapse. Similar to their HSC counterpart, LSCs reside in a highly specialized microenvironment referred to as the “niche”. Bidirectional interactions between leukemic cells and the microenvironment favor leukemic progression at the expense of healthy hematopoiesis. Within the niche, LSCs are thought to be protected from genotoxic insults. Improvement in our understanding of LSC gene expression profile and phenotype has led to the development of prognosis signatures and the identification of potential therapeutic targets. In this review, we will discuss LSC biology in the context of their specific microenvironment and how a better understanding of LSC niche biology could pave the way for new therapies that target AML.

## Introduction

Acute myeloid leukemia (AML) is the most common type of acute leukemia in adults. AML is characterized by the clonal proliferation of abnormal hematopoietic progenitors leading to blood and bone marrow infiltration and consequently hematopoietic failure (1). Over the past decades, intensive research has significantly improved our understanding of AML biology, highlighting the role of clonal evolution and identifying potential therapeutic targets based on recurrent molecular abnormalities (2, 3). However, therapeutic progress has been limited (4). Despite a promising initial response to intensive chemotherapy, relapse occurs in the majority of patients and prognosis remains poor with a long-term overall survival of 40-50% in patients younger than 60 years old (5–8). In older patients not able to endure intensive chemotherapy, therapeutic options are limited, and long-term overall survival remains low at 15% (9, 10).

Leukemic stem cells (LCSs), also sometimes referred to as leukemic initiating cells, were first described 25 years ago, when Lapidot et al. showed that a small subset of leukemic cells could be transplanted and give rise to leukemia in immunocompromised recipient mice (11). The same group latter identified the CD34^pos^CD38^neg^ phenotype as a way to enrich the LSC population. Similar to normal hematopoietic stem cells (HSCs), LSCs are able to differentiate and self-renew suggesting a leukemic hierarchy (12–16).

Like their normal counterpart, LSCs reside in the bone marrow in a specialized microenvironment termed “niche”. Schofield first described the concept of niche in 1978 and defined it as a limited specific anatomical site where stem cells could be maintained, undergo self-renewal, and where differentiation is inhibited (17). Over the past 20 years, the development of transgenic mice and the improvement of imaging techniques has led to several breakthrough discoveries suggesting that the bone marrow microenvironment plays a central role in normal and pathological hematopoiesis (18). Within the niche, LSCs are thought to be protected from chemotherapy (19–22). Therefore, targeting the LSCs niche represents a promising option to cure AML.

## Leukemic Stem Cells Ontogeny And Phenotype

The concept of LSCs is based on the idea that a small subset of cells is able to continually replenish the bulk of leukemic cells. Leukemic stem cells are defined by their capacity to self-renew, incompletely differentiate, and reinitiate leukemia upon serial transplantation in immunocompromised mice (11, 23). Initially thought to originate from the healthy HSC compartment, recent studies have shown that LSCs may instead emerge from committed progenitors (24, 25). Most of human AMLs have at least two molecularly hierarchically ordered distinct LSCs populations (24). Interestingly, the more mature LSC population most closely mirrors normal granulocyte-macrophages progenitors (GMP) whereas the immature LSC population is functionally similar to lymphoid-primed multipotent progenitors (LMPPs). Leukemia originates from the acquisition of driver mutations by HSC or early progenitors (26–28). Identification of clonal hematopoiesis of indeterminate potential (CHIP) has recently generated a significant interest (29). The sequential acquisition of mutations in HSCs and progenitors over a lifetime is suspected to favor hematological malignancies. However, given the high frequency of CHIP in the general population, the exact significance of these mutations and implication in leukemogenesis still needs clarification. To add more complexity, LSCs ontogeny seems to be reversible as opposed to the previously accepted idea that LSCs unidirectionally differentiate into mature AML cells. Indeed, PU.1 gene suppression in differentiated AML-derived cells has been shown to revert AML cells to an immature, clonogenic leukemogenic state (30).

Following the pioneering work done by John Dick’s group, showing that LSCs are enriched within the CD34^pos^CD38^neg^ fraction, several surface markers have been described. Indeed, studies showed that when compared to normal HSCs, LSCs displayed a higher expression of CD25 (31), CD32 (31), CD44 (32), CD96 (33), CD123 (34–36), GPR56 (37), C-type lectin-like molecule-1 (38), IL1RAP (Interleukin 1 Receptor Accessory Protein) (39, 40), N-cadherin, and Tie2 (41). However, a high intra and inter-patients’ heterogeneity prevents the use of a single surface marker to easily isolate LSCs.

## The Healthy Hematopoietic Niche

Hematopoietic stem cells reside in a highly specialized microenvironment or niche within the bone marrow (18). Cellular and molecular interactions between niche constituents and HSCs tightly control their self-renewal, proliferation, and differentiation properties. The development of reporter mice and the improvement of imaging techniques has led to a better understanding of the niche since the concept was first proposed in 1978 (17). Studies have identified several cell populations, sometimes redundant, implicated in homeostatic and pathologic hematopoiesis. Similar to the heterogeneity of the hematopoietic system, niche cells are also highly heterogeneous (42–46).

Early studies have suggested a major role of osteoblasts in hematopoiesis by showing hematopoietic stem and progenitor cells (HSPCs) and osteolineage cells in close proximity at steady state and after bone marrow transplantation, additionally osteoblasts have the capacity to support HSPCs *in vitro* (47–50). Other studies showed a correlation between the number of osteoblasts and Lin^neg^Sca1^pos^c-Kit^pos^ HSPCs (51, 52). However, the specific genetic deletion in osteoblast of two key cytokines required for HSC maintenance, *stem cell factor* (*Scf*) and *CXC-chemokine ligand 12* (*Cxcl12)*, did not have a major effect on HSCs (53–55). In addition, 3-D imaging of the bone marrow revealed that HSCs were preferentially localized close to the vascular network but not to the endosteal surface (56, 57). However, osteolineage cells form a niche for early lymphoid progenitors (53, 54, 58), and are implicated in the development and progression of several hematological malignancies like leukemia (54, 58–61).

The identification of the SLAM cell surface markers allowed the imaging of purified HSCs in their native niche (62). This study and others revealed the close proximity of HSCs and blood vessels suggesting the existence of a vascular niche composed by different types of blood vessels and associated perivascular cells (18). Bone marrow mesenchymal stem cells (BM-MSCs) represent a rare and heterogeneous population of stromal cells characterized by their ability to self-renew and differentiate into osteoblasts, chrondrocytes and adipocytes (63). In the bone marrow, MSCs are located around the blood vessels where they closely interact with HSCs and support hematopoiesis. The development of new transgenic mice models led to the identification of several MSC subsets with significant overlap between the different populations identified (53, 55, 64–68). BM-MSCs are major sources of key niche factors important for the maintenance, proliferation and retention in the mouse bone marrow of HSCs (69). Deletion of *Scf* or *Cxcl12* in stromal cells directly affects HSC number and localization (67, 70, 71). Recent single cell RNA sequencing-based studies have confirmed the high heterogeneity among stromal cells in the bone marrow in particular within the MSC compartment at an unprecedented resolution (42, 44, 45).

The bone marrow is highly vascularized which provides nutrients and oxygen and furthermore allows HSCs and newly generated hematopoietic cells to leave the bone marrow and circulate throughout the body. Bone marrow vascularization is composed of thin-walled arterioles paralleled to the long bone axis and mostly closed to the endosteal region. Arteriolar vessels are connected to the dense network of highly branched sinusoids by type-H vessels at the proximity of the bone (72). Endothelial cells are also key regulators of HSC maintenance and function, and most HSCs localize within 5µm of a bone marrow vessel (56, 62). Indeed, endothelial cells express several factors that regulate HSC function such as SCF, CXCL12, and Notch ligands among others. Depletion of these factors has a dramatic effect on HSC number at steady state and hematopoietic recovery following myeloablative treatment (53–55, 73, 74).

The nervous system plays a crucial role in bone and bone marrow homeostasis (75). Whereas parasympathetic fibers only innervate the compact bone, the bone marrow cavity is innervated by both sympathetic and sensory nerves (76, 77). Although sympathetic nerves do not regulate HSC directly, they are important regulators of HSC mobilization from the bone marrow in response to G-CSF (78). HSCs are also released into the circulation in a circadian manner in response to adrenergic signals from the sympathetic nervous system (SNS) that regulate the synthesis of MSC derived CXCL12, critical for the retention of HSCs inside the bone marrow (65, 78, 79). Interestingly, nociceptive nerves collaborate with the SNS in HSC maintenance and G-CSF-induced mobilization *via* the secretion of calcitonine gene-related peptide (80). Bone marrow neuropathy observed in aging or after the administration of genotoxic drugs induced a profound remodeling of the HSC niche and affected bone marrow regeneration (81–83). Non-myelinating Schwann cells are also involved in HSCs maintenance by converting the latent Transforming Growth Factor β (TGFβ) into the active form inducing HSCs quiescence (84).

In addition to bone marrow stromal cells, healthy HSCs are also directly and indirectly regulated by their own hematopoietic progeny including megakaryocytes, macrophages, regulatory T cells, neutrophils and other myeloid cells, reviewed elsewhere (18).

## The Leukemic Niche

Although the exact location of LSCs within the bone marrow niche still needs to be clarified, it is now clear that the microenvironment plays a role in leukemogenesis and that leukemic cells can also alter the bone marrow at the expense of physiological hematopoiesis.

### A Potential Role of the Microenvironment in Leukemogenesis

Leukemogenesis was long regarded as a cell autonomous process. This dogma was challenged by the early description of donor cell derived leukemia in bone marrow transplanted patients (85). These observations supported the “seed and soil” theory proposed by Paget in 1889 who suggested that tumor metastasis required favorable interactions between tumor cells (the “seed”) and their microenvironment (the “soil”) (86). The role of non-hematopoietic cells in leukemogenesis was first demonstrated by the development of transgenic mice and the capacity to delete genes in a cell-specific manner. In the context of hematological malignancies, the proof of concept came from the description of a myeloproliferative disorder induced by deregulated expression of Jagged 1 in IκBα deficient hepatocytes. In contrast, mice with a conditional deletion of IκBα specifically in the myeloid lineage did not develop any myeloproliferative neoplasm (MPN) (87), suggesting that premalignant hematopoietic disorders can be initiated by nonhematopoietic cells. Walkley, et al. demonstrated the role of the retinoic acid receptor-γ (RARγ) in niche-driven MPN. Mice deficient in RARγ developed a MPN-like phenotype even when transplanted with wild-type cells (88). The same group investigated the role of the retinoblastoma protein (RB) in hematopoiesis and demonstrated that the deletion of *Rb* induced a MPN-like phenotype only when deleted in both the hematopoietic and non-hematopoietic compartments (89). These studies support the role of the interaction between hematopoietic cells and their microenvironment in the development of hematological malignancies.

Bone marrow MSCs play a central role in the regulation of HSCs during homeostatic hematopoiesis while also involved in the development of myelodysplasia and leukemia. Indeed, specific deletion of the gene encoding Dicer 1, an enzyme involved in micro-RNA processing in osteoprogenitors induces myelodysplasia and sporadic secondary leukemia (59). This phenotype was not observed when *Dicer1* was deleted in the hematopoietic cells demonstrating that the myelodysplasia was environmentally induced. Deletion of *Dicer1* induced the downregulation of *Sbds*, a gene mutated in Schwachman-Bodian-Diamond syndrome, which is a rare human disease characterized by bone marrow failure and a predisposition to leukemia. Specific deletion of *Sbds* in MSCs induced mitochondrial dysfunction, oxidative stress, and activation of the DNA damage response in HSPCs ultimately impairing hematopoiesis and favoring leukemogenesis (90). This effect is a consequence of the secretion of the pro-inflammatory molecules, S100A8 and S100A9, by MSCs. Conditional expression of a mutated form of *Ptpn11*, the gene encoding for the protein tyrosine phosphatase SHP2, in MSCs and osteoprogenitors also induced a MPN-like phenotype (91). To further support the role of the osteolineage compartment in leukemogenesis, activating mutation of beta-catenin in osteoblasts induced AML by activation of Notch signaling in HSPCs (92). By contrast, the defective activation of Notch in the microenvironment leads to myeloproliferative disease (93). This effect is attributed to a Notch-dependent repression of the micro-RNA miR-155, regulating the inflammatory state of the bone marrow niche (94).

Healthy hematopoiesis is the consequence of close and highly regulated interactions between HSPCs and their microenvironment. Overall, cumulative evidence suggests that niche constituents can also drive hematopoietic malignancy.

### Remodeling of the Hematopoietic Niche by Leukemic Cells

**Figure 1:**
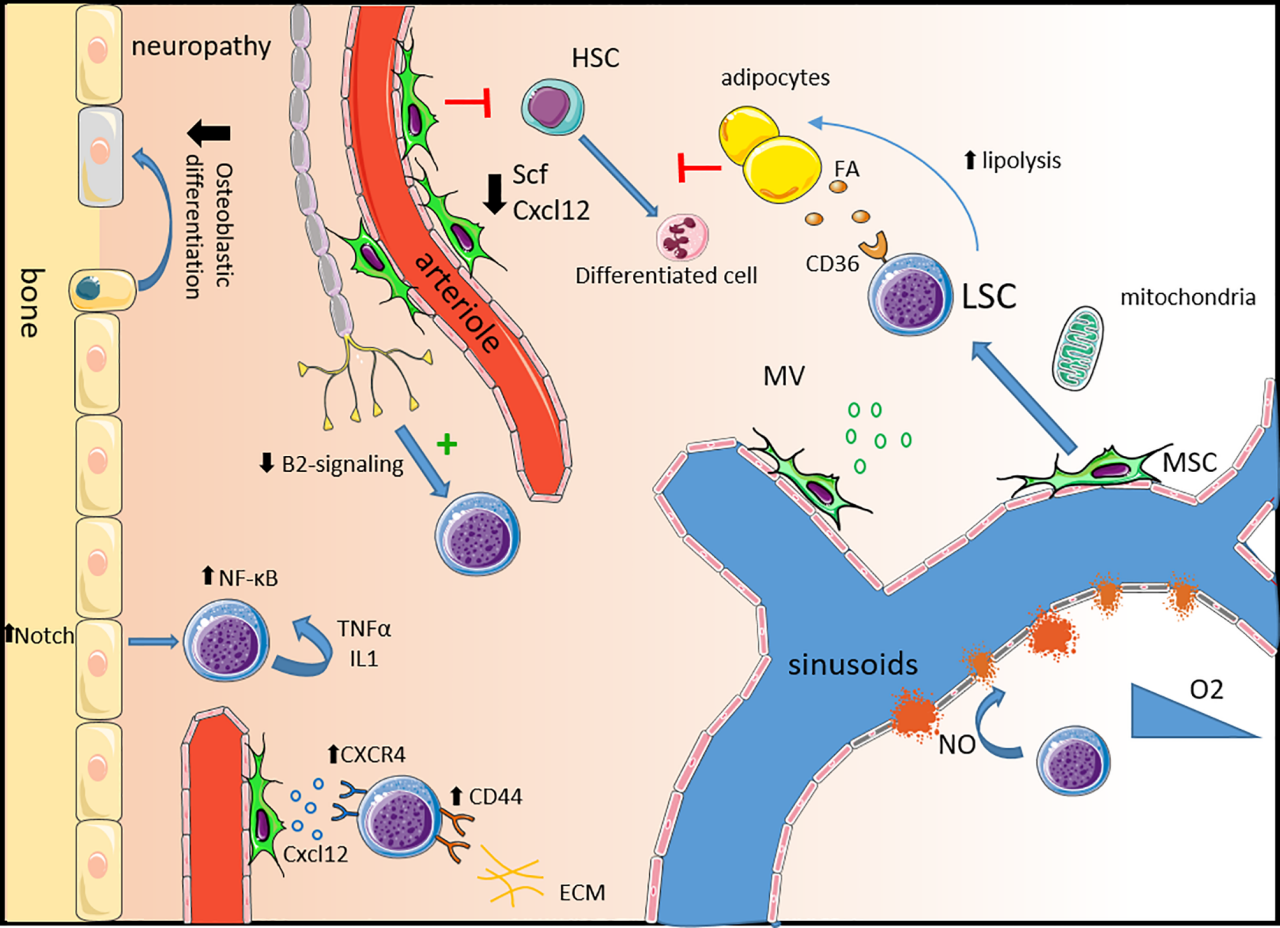
Remodeling of the healthy niche into a permissive leukemic niche. *Neuropathy:* Leukemic progression is associated with sympathetic neuropathy. Loss of β2-adrenergic signaling directly promotes leukemic progression and triggers the expansion of MSCs primed for osteoblastic differentiation but with a defect in terminal maturation leading to a reduction in mineralized trabecular bone. *Mesenchymal stem cells*: In leukemia, MSCs are dysfunctional expressing lower levels of key healthy HSC niche factors such as *Scf* and *Cxcl12* impairing healthy hematopoiesis. LSCs express high levels of the CXCL12 receptor CXCR4 and other adhesion molecules such as CD44 and VLA-4 to usurp the adhesion mechanisms of healthy HSCs. MSC also contribute to LSC survival by the production of microvesicules and *via* mitochondria transfer, providing energy support. *Alteration of the vascular niche:* The expression of VEGF in the leukemic niche induces an increase in vascular density and the production of NO by endothelial cells increases vascular leakiness contributing to hypoxia. In leukemia, endosteal blood vessels are more disrupted than the central bone marrow ones. *Adipocytes:* Leukemic cells support their own metabolism and survival by stimulating lipolysis which fuels fatty acid oxidation in chemotherapy resistant LSCs expressing the fatty acid transporter CD36. *Inflammatory niche*: Activation of Notch signaling in osteolineage cells leads to the activation of the NF-κB pathway in leukemic cells supporting their survival and proliferation. An autocrine secretion of pro-inflammatory molecules like IL-1 and TNF-α also activates the NF-κB pathway. HSC, hematopoietic cells; SCF, stem cell factor; FA, fatty acid; LSC, leukemic stem cell; MSC, mesenchymal stem cell; MV, microvesicule; NO, nitric oxide; ECM, extracellular matrix.

As our knowledge of the normal hematopoietic niche improved in the past 20 years, the role of the microenvironment in leukemia development captured the attention of the field. Leukemic cells can remodel the niche creating a favorable microenvironment at the expense of the normal hematopoiesis (**Figure 1**) (95). Imaging studies in mice have shown that chemotherapy resistant human LSCs primarily home to and engraft close to the endosteal region where they closely interact with different microenvironmental structures (19).

The bone marrow vascularization is altered in AML with an increased micro-vessel density consequence of the production of pro-angiogenic factors like vascular endothelial growth factor (VEGF) (96–99). AML progression induces the production of nitric oxide (NO) which increases vascular permeability and maintains overall hypoxia (100). Interestingly AML leads to a differential remodeling of vasculature in central and endosteal regions (101). A preferential disruption of the endosteal blood vessels leads to progressive remodeling of the endosteal stroma and the progressive loss of stromal cells. Inhibition of the AML-driven vascular remodeling was shown to improve chemotherapy efficiency in mice (100, 101).

Leukemic cells can reprogram MSCs to create a pro-tumoral niche. MSCs reprogramming can occur following direct cell-to-cell contact, *via* secreted factors, or *via* exosomes (102–104). In addition, human MSCs isolated from AML patients (AML-MSC) displayed *in-vitro* reduced proliferative potential and increased levels of apoptosis (105). Compared to MSCs isolated from healthy donors, AML- MSCs have a lower expression of several niche factors such as SCF, THPO, ANGPT1, VCAM1 and BMI1 (106). In mice, MSCs support AML cells by transferring mitochondria to provide additional energy (107, 108). This transfer is enhanced by some chemotherapies and provides a survival advantage to leukemic blasts and LSCs. This transfer occurs through AML-derived nanotubes. Study in mice showed that superoxide produced by AML cells NADPH oxidase-2 (NOX2) stimulates the nanotubes formation in MSCs. Interestingly, inhibition of NOX2 was able to prevent mitochondrial transfer and improved survival in a xenograft model (107). MSCs also help LSCs to cope with increased reactive oxygen species (ROS) levels, consequence of the mitochondrial transfer by providing increased bioenergetics and detoxifying enzymes (109). Furthermore, MSCs protect AML from chemotherapy through increased Notch and Wnt signaling and inhibition of apoptosis (110–113). Dysregulation of the cytokine profile is suspected to create a pro-tumoral niche in AML (114, 115). LSCs reside in a pro-inflammatory environment known to favor LSCs survival and proliferation. As opposed to normal HSCs and differentiated blasts, LSCs exhibit constitutive NF-κB activity. This activity is partly the consequence of an autocrine tumor necrosis factor-α (TNF-α) secretion, formed by an NF-κB/TNF-α positive feedback loop (116). Activation of Notch signaling also contributes to the activation of the NF-κB pathway (111). Similarly, LSCs aberrantly express the co-receptor for interleukine-1 (IL-1), IL1RAP. Downregulation of IL1RAP inhibits the clonogenic activity of AML cells and leads to increased apoptosis (39). Interestingly, LSCs express IL-1 suggesting another pro-inflammatory autocrine loop. Within the leukemic niche, cytokines can be produced by either immune or leukemic cells. Several cytokines and soluble factors have been shown to affect leukemic cells survival and growth *in-vitro* (117). While pro-inflammatory cytokines such as IL-1β, GM-CSF, IL-3, TNF-α seem to promote AML cells growth, anti-inflammatory molecules such as IL-1Rα, TGF-β and IL-10 have an inhibitory effect (117–119). The function of a specific cytokine is dependent on multiple complex molecular interactions within the microenvironment. Therefore, despite a major improvement in our understanding over the past decade, further studies are needed to clarify the cytokine network in AML.

Adipocytes are classically considered negative regulators of normal hematopoiesis (120). However, this negative action seems to depend on adipocytes anatomical location. Indeed, adipocytes in the active red bone marrow support blood regeneration and myelo-erythroid maturation (121, 122). In the context of AML, leukemic cells repress bone marrow adipocyte maturation impairing myelo-erythoid differentiation (122). Leukemic cells induce the lipolysis of triglyceride to free fatty acids supporting their proliferation and survival (123). Interestingly, outside the bone marrow, gonadal adipose tissue represents a reservoir for LSCs. Within this adipose tissue, leukemic cells create an inflammatory environment triggering lipolysis and the released of fatty acids that fuel LSCs expressing the fatty acid transporter CD36, contributing to chemo-resistance (124).

The sympathetic nervous system is a critical regulatory component of the bone marrow microenvironment that controls the plasticity of bone marrow stromal cells under homeostatic conditions (78, 79, 125). Aging, a condition associated with myeloid biased hematopoiesis and an increased risk of myelodysplastic syndromes and leukemia is associated with sympathetic neuropathy and decreased β3-adrenergic signaling (82, 83). In a MLL-AF9 mouse model, AML infiltration induced sympathetic neuropathy which further promoted AML (60). This neuropathy was associated with an expansion of Nestin-GFP^pos^ MSCs primed for osteolineage differentiation, and HSC exhaustion. Loss of β2-adrenergic signals directly promotes an expansion of LSCs expressing the β2-adrenergic receptor. Studies using primary AML cells from patients showed that leukemic cells altered adipogenesis in favor of osteolineage differentiation (122, 126). However, sympathetic neuropathy impairs terminal osteoblastic lineage differentiation leading to a reduction in mineralized bone density (60). Sympathetic neuropathy was also induced by the pro-inflammatory environment observed in a JAK2^V617F^ MPN mouse model (127). In this context, Nestin-GFP^pos^ MSCs are reduced, which in turn led to the expansion of altered HSPCs and disease progression.

Similar to their healthy counterpart, LSC localization is dependent on the expression of cytokines and adhesion receptors. Leukemic cells adhere to the bone marrow through three main receptors: CXCR4, Very Late Antigen-4 (VLA-4) and CD44 (128). The high expression of these adhesion molecules facilitates the homing and retention of leukemic cells in the niche impairing chemosensitivity (32, 129–131). In addition, interactions between VLA-4 expressed by leukemic cells and VCAM1 expressed at the surface of BM-MSC mediates chemoresistance *via* activation of the NF-κB pathway in stromal cells (20).

## Leukemic Stem Cells: A Therapeutic Opportunity

### LSCs as a Prognostic Marker

Patients with AML are treated according to a risk stratification aiming to identify the patients with low, intermediate, and high risk of relapse based on the disease characteristics at diagnosis (9). Since LSCs have been implicated in treatment resistance and relapse, quantification of the LSC pool could be an additional prognostic factor beside the traditional genetic and molecular abnormalities. As we discussed before, a clear definition of the LSC phenotype does not exist, and different approaches have been used to estimate the LSC pool in patients. Using flow cytometry, Zeijlemaker W. et al. showed that CD34-positive AML blasts were associated with an increased incidence of relapse compared to CD34-negative AML (132). More recently, the prognostic impact of LSC frequency defined by the CD34^pos^CD38^neg^ phenotype combined with minimal residual disease (MRD) evaluation was demonstrated in a prospective study (133). High level of CD34^pos^CD38^low^/CD123^pos^ blasts at diagnosis is predictive of an adverse outcome (134). Interestingly, a recent study performed in older AML patients showed that this predictive impact is only seen in patients treated by intensive chemotherapy but not by hypomethylating agents (36). Leukemic stem cells frequency seems to be correlated with a lower white blood cell count, an adverse cytogenetic risk, and less frequent NPM1 mutation (36, 135).

Stem cell gene expression signatures have been shown to have a prognostic impact in AML, also highlighting the potential role of leukemia stemness in treatment response (25). Based on this observation, a 17 genes score (LSC17) that compared the gene expression profiles between 138 LSC^pos^ and 89 LSC^neg^ isolated from 78 AML patients was developed (136). A high score is associated with a poor outcome after standard treatment including HSC transplantation (136). The LSC17 was recently challenged by the newly developed AML prognostic score (APS), a 16 gene expression signature score, derived from RNA-sequencing and whole exome sequencing results (137). Interestingly, the authors hypothesized that APS can outperform the LSC17 because of its capacity to capture signal from the microenvironment.

### How to Target the Leukemic Stem Cell Niche

**Figure 2:**
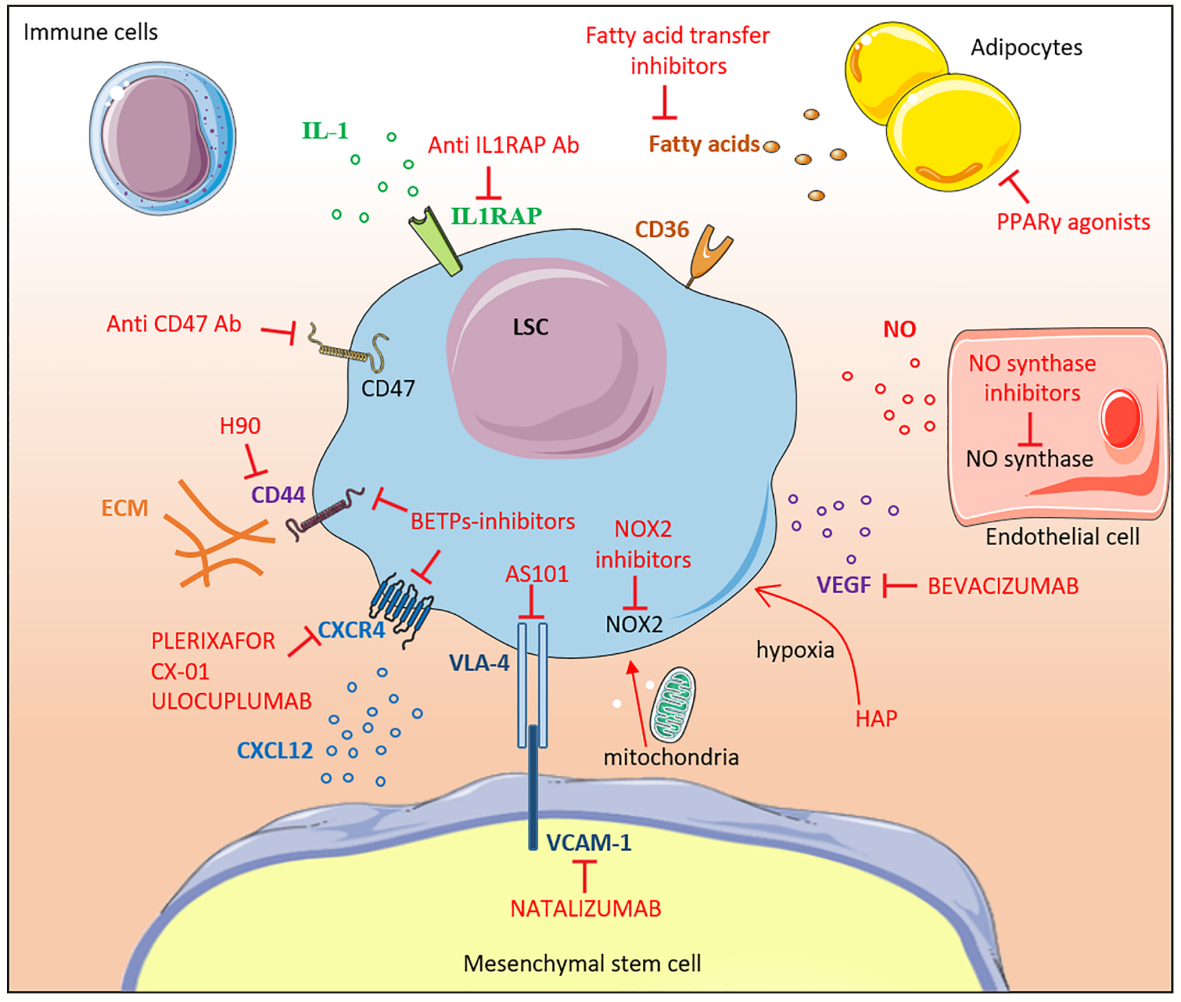
Therapeutic targeting of the leukemic niche. The different molecular interactions between LSCs and the bone marrow niche constituents are shown. Inhibitors are labeled in red. Most of the drugs shown in the figure are under pre-clinical or early clinical development. IL-1, interleukine-1; Ab, antibody; CD, cluster of differentiation, FA, fatty acid; LSC, leukemic stem cell; MSC, mesenchymal stem cell; NOX2, NADPH oxidase 2; NO, nitric oxide; ECM, extracellular matrix; VEGF, vascular endothelial growth factor; HAP, Hypoxia-activated prodrugs; PPARγ, Peroxisome Proliferator-activated Receptor gamma; VCAM-1, Vascular Cell Adhesion Molecule-1; BETPs, Bromodomain Extra-Terminal Protein.

Compared with other hematological malignancies, therapeutic progresses have been limited in AML highlighting the need for new strategies. The microenvironment shelters LSCs, protects them from genotoxic drugs and therefore represents a possible cause of treatment failure and relapse. Different strategies have attempted to target the LSC-niche interactions and several studies are currently ongoing (**Figure 2**). LSCs can also be directly targeted based on their phenotypic and functional differences compared to healthy HSCs. These strategies are beyond the scope of this article and have been reviewed elsewhere (31, 138–140).

#### Adhesion Molecules

Adhesion molecules maintain LSCs in the hypoxic niche protecting them from cycling-dependent chemotherapies. Targeting adhesion molecules aims to mobilize LSCs out of their protective niche in order to expose them to chemotherapy. LSCs express the receptor CXCR4 and migrate in response to CXCL12 (141). Moreover, high levels of CXCR4 expression are associated with relapse and poor overall survival in patients (142). Plerixafor, a potent inhibitor of CXCR4, is currently used in association with G-CSF to induce HSCs mobilization (143). In an acute promyelocytic leukemia murine model, treatment with plerixafor in combination with cytarabine and daunorubicine improved chemosensibility and overall survival (144). Since this early study, plerixafor has been tested in phase I-II studies, in combination with various chemotherapies and hypomethylating agents with promising results (145–147). Other CXCR4-CXCL12 axis inhibitors are under clinical development like CX-01, BL-8040 and ulocuplumab. These drugs showed encouraging results in combination with chemotherapy in phase I-II studies (148–151). However, larger phase III studies are needed to confirm the benefit and the exact place of the CXCR4-CXCL12 axis inhibition in AML treatment strategy.

Bromodomain and extra-terminal domain-containing (BET-containing) proteins (BETPs)-inhibitors, can also target adhesion molecules. Sustained degradation of BETPs induced the downregulation of CXCR4 and CD44 expression, decreased the LSC population, and improved overall survival in a patient-derived xenotransplantation model (152). Importantly, BETPs inhibition significantly reduced the number of LSCs when used alone or in combination with chemotherapy. CD44 represents an exciting target since it is differentially expressed between LSCs and normal HSCs (130, 131). Administration of H90, a monoclonal antibody directed to CD44, in immunocompromised mice transplanted with human AML reduced the leukemic burden. Interestingly, H90 seemed to specifically target the LSCs population since no leukemia was observed in serially transplanted mice (32).

#### Vascularization Remodeling and Hypoxia

VEGF was early identified as a promising target given its pro-angiogenic and anti-apoptotic effects on leukemic cells (153). However, results of clinical studies using bevacizumab, a humanized recombinant monoclonal antibody directed against VEGF have proven disappointing (154, 155). A recent study in mice suggests that inhibition of NO production by endothelial cells could restore the normal vascularization and improve response to cytarabine (100). Targeting NO production by inhibiting the NO synthase could therefore represent a new therapeutic target. The niche represents a hypoxic environment that maintains LSCs in a quiescent state. Moreover, hypoxia inducible factor-1 (HIF-1α) expression induced by hypoxia upregulates CXCR4 expression at the membrane surface of LSCs (19). However, the exact impact of HIF-1α inhibition is still debated (156, 157). Another way to target the hypoxic microenvironment is to use hypoxia-activated prodrugs (HAPs) (158) specifically designed to form cytotoxic agents under hypoxic conditions while limiting the toxicity on normal tissues. Evofosfamide (also known as TH-302) is a 2-nitroimidazole-linked prodrug. *In vitro*, evofosfamide treatment promotes a dose- and hypoxia-dependent apoptosis and cell death in AML cells. Interestingly, in a xenograft model, evofosfamide reduces LSC pool with limited toxicity on normal hematopoiesis (159, 160). However, a phase I study conducted in 49 patients with advanced leukemia showed disappointing results with an overall response rate of only 6% only (161). Other HAPs are currently under development.

#### Cytokines and Soluble Factors

Targeting the pro-inflammatory environment represents another interesting strategy considering the importance of cytokines like IL-1, IL-6 and TNFα for LSC survival and proliferation. IL-1 and IL-6 inhibitors are already commercially available for the treatment of autoimmune disease and cytokine released syndromes (162, 163). It would be interesting to test these inhibitors in combination with chemotherapy even if caution is needed regarded the risk of infections. Given the higher expression of IL1RAP at the surface of LSCs compared with normal HSCs, targeting IL1RAP is an attractive option. Indeed, in a preclinical study, targeting IL1RAP using a monoclonal antibody induced selective killing of AML CD34^pos^CD38^pos^, and CD34^pos^CD38^neg^ cells both *in vitro* and in a xenograft model (164).

Since leukemic cells trigger lipolysis and use fatty acids as a source of energy, targeting the adipose tissue represents another possible strategy. Studies in mice have shown that restoring normal adipocyte maturation using PPARγ agonists inhibits leukemic growth. Similarly, inhibiting fatty acids transfer to leukemic cells improved survival in a xenograft model (123). However, further studies in human are warranted.

## Conclusion

According to the cancer stem cell theory, LSCs sit at the top of the hierarchy and are the source of the more differentiated leukemic blasts. Even if these cells represent an attractive target, eradicating LSCs is highly complex, notably due to the lack of specific markers. AML is associated with a remodeling of the hematopoietic niche where HSCs and LSCs reside, however, modifications of the microenvironment also contribute to leukemia development at the expense of normal hematopoiesis. Since the first description of LSCs more than 25 years ago, our understanding of this small subset of leukemic cells has greatly improved with the identification of potential therapeutic targets paving the way for the development of new treatment strategies in a still deadly disease.

## Author Contributions

TM and SP conceptualized and finalized the manuscript. All authors contributed to the article and approved the submitted version.

## Funding

TM is supported by the “association pour le développement de l’hématologie oncologie/ADHO”.

## Conflict of Interest

The authors declare that the research was conducted in the absence of any commercial or financial relationships that could be construed as a potential conflict of interest.

## Publisher’s Note

All claims expressed in this article are solely those of the authors and do not necessarily represent those of their affiliated organizations, or those of the publisher, the editors and the reviewers. Any product that may be evaluated in this article, or claim that may be made by its manufacturer, is not guaranteed or endorsed by the publisher.
